# Case Report: Neurolymphomatosis of the peripheral nerve as a presentation of relapsed pediatric high-grade B cell lymphoma

**DOI:** 10.3389/fonc.2025.1477848

**Published:** 2025-05-01

**Authors:** Evelyn Ruoyun Lu, Wai Lan Yeung, Jeffrey Ping Wa Yau, King Sun Chu, Elaine Yee Ling Kan, Alan Kwok Shing Chiang

**Affiliations:** ^1^ Department of Pediatrics and Adolescent Medicine, Hong Kong Children’s Hospital, Hong Kong, Hong Kong SAR, China; ^2^ Department of Pediatrics and Adolescent Medicine, Li Ka Shing Faculty of Medicine, The University of Hong Kong, Hong Kong, Hong Kong SAR, China; ^3^ Department of Nuclear Medicine, Queen Elizabeth Hospital, Hong Kong, Hong Kong SAR, China; ^4^ Department of Radiology, Hong Kong Children’s Hospital, Hong Kong, Hong Kong SAR, China

**Keywords:** neurolymphomatosis, peripheral neuropathy, high grade B cell lymphoma, neuropathy, pediatric lymphoma

## Abstract

Neurolymphomatosis (NL) of the peripheral nerve in pediatrics has never been reported, and ultrasonography (USG) as an investigation modality is rarely used. We report a case of an 11-year-old boy with stage 4 mature high-grade B cell lymphoma who presented with a two-week history of right ulnar neuropathy and left sciatic neuropathy one month after the completion of frontline chemotherapy. This case report illustrates the rare presentation of NL in lymphoma relapse and hopes to emphasize how early identification of this diagnosis and timely treatment are essential. Moreover, this case points out how USG, in the context of palpable mass and localized neurological signs, could facilitate a diagnosis of NL.

## Introduction

Neurolymphomatosis (NL) is a local invasion of cranial or peripheral nerves by lymphoma cells. NL, as a presentation in pediatric lymphoma, is very rare. Three reported pediatric cases of NL involved cranial nerves, nerve roots, and cauda equina instead of peripheral nerves (1–3). Diagnostic dilemmas for neurolymphomatosis are often encountered as they mimic the presentation of other causes of neuropathy, such as infectious, inflammatory, or paraneoplastic. There is no consensus on the best investigation modality to diagnose NL. Here, we report an 11-year-old boy with relapsed high-grade B cell lymphoma who presented with a picture of ulnar and sciatic neuropathy and was subsequently diagnosed with neurolymphomatosis of the peripheral nerve by ultrasonography (USG).

## Background

An 11-year-old boy was first diagnosed with stage 4 high-grade B cell lymphoma upon presentation with two weeks’ history of fever and malaise as well as hepatosplenomegaly. Lactate dehydrogenase level was two times above the upper limit standard of institutional adult value at 732 IU/L. Diagnostic CT and PET-CT scans showed nodal involvement on both sides of the diaphragm, extranodal lesions at the scalp, left second to third ribs, and right ilium. There was central nervous system involvement evidenced by malignant cells detected in cerebrospinal fluid (CSF) and bilateral bone marrow infiltration (≥25% lymphoma infiltrates). The lymphoid cells in bone marrow aspirates were positive for CD19, CD20, CD22, CD38, and CD79b with monotypic kappa light chain expression by flow cytometry. Cytogenetics showed translocation of 3q27 and 14q32. Rearrangement of BCL6 and IGH was confirmed by fluorescence *in situ* hybridization. MYC and BCL2 rearrangements were not detected. In summary, our patient was diagnosed to have mature high-grade B cell lymphoma with central nervous system disease.

He was categorized into the highest-risk group. He received chemotherapy in the C3 arm of the Inter-B-NHL ritux 2010 protocol, which comprises six cycles of LMB-based multiagent B-cell-directed chemotherapy with six doses of rituximab (4). After induction and consolidation chemotherapy cycles were completed, reassessment CT scans and bone marrow examination showed complete remission of the disease. He proceeded to complete two maintenance chemotherapy cycles. PET-CT scan performed three weeks after completion of chemotherapy showed remission in all primary lesions and bone marrow except for increased fluorodeoxyglucose (FDG) activity over the right testis and anterior right mid-arm. Although physical examination showed no palpable scrotal mass, subsequent USG of the scrotum revealed possible lymphomatous infiltration of both testes.

**Figure 1:**
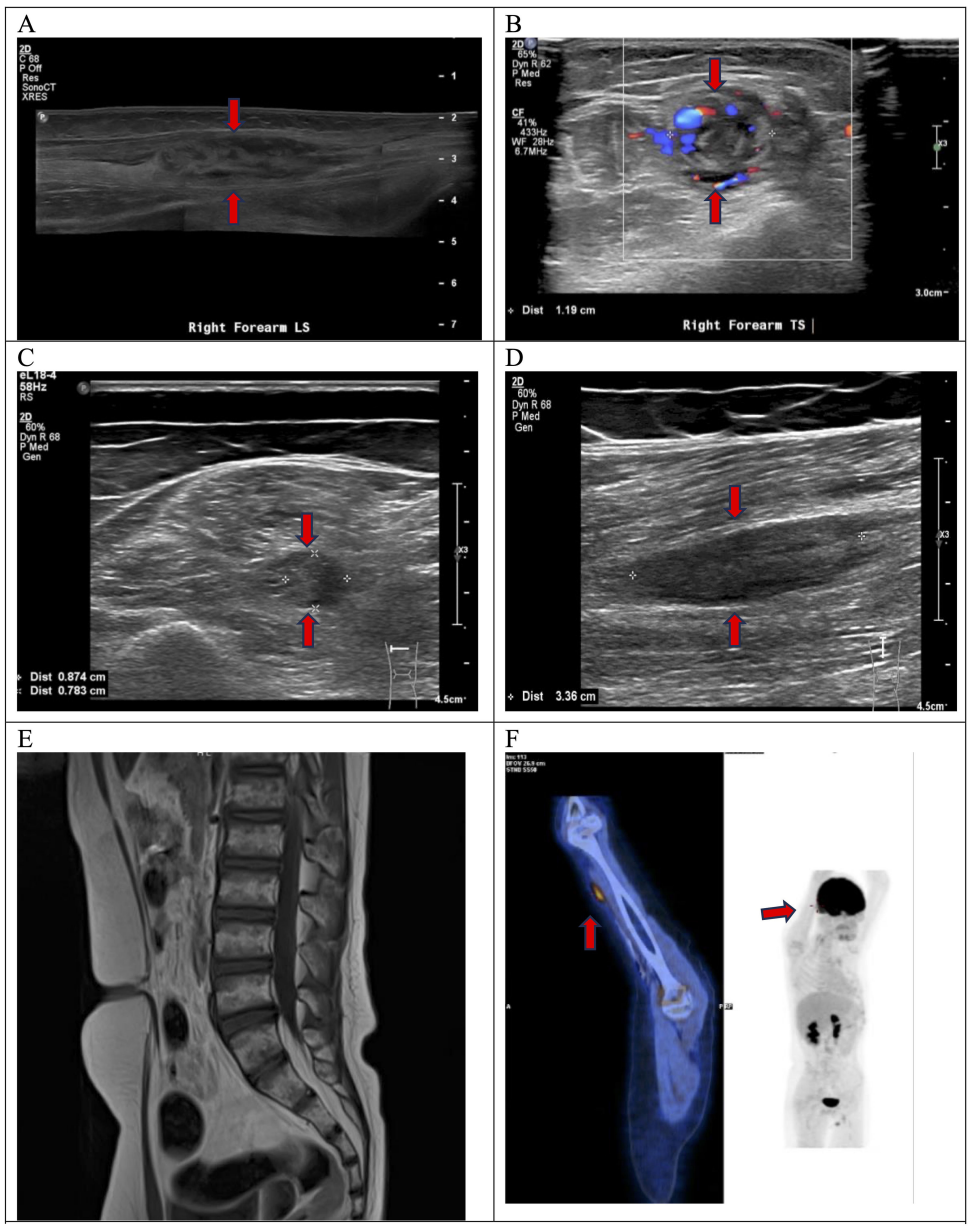
At the right mid-forearm, USG demonstrated an elongated heterogeneous lesion along the right ulnar nerve **(A)**, measuring 1.3x1.2x5.8cm in size with increased intralesional color doppler signal seen on transverse view **(B)**. At the left mid-thigh level, transverse view showed an abnormal fusiform swelling of medial component of the left sciatic nerve affecting the tibial trunk **(C)**. Longitudinal view confirmed that the swelling was intraneural. It measured 0.9x0.8x3.4cm in size **(D)**. MRI spine showed no compression of cauda equina **(E)**. FDG PET-CT showed increased FDG avidity at anterior right mid-arm with SUV max 3.1 **(F)**.

One month after the completion of chemotherapy, the patient developed a two-week history of persistent pain and numbness over the right fifth finger and a tender lump over the right wrist. Examination of the right hand showed atrophy of the hypothenar muscle and weakness of the abductor digiti minimi, first dorsal interosseous muscle, third and fourth lumbricals, fourth and fifth flexor digitorum profundus, and flexor carpi ulnaris. The sensation was decreased over the medial part of the right hand. Two palpable masses, the largest at 1.5 cm, were felt at the right mid-arm and forearm. The overall picture was consistent with right ulnar neuropathy. USG of the right upper limb showed elongated heterogeneous lesions arising from the right ulnar nerve at the right mid-arm and forearm (**Figures 1A, B**).

He also had a gradual onset of pain over the left calf and posterior thigh and could not bear weight or walking. He had no sphincter symptoms. Examination showed absent movement from the left ankle and below, absent ankle reflex, and reduced sensation over the left lateral leg, dorsum, and sole. These clinical findings indicated left sciatic neuropathy. USG of the left lower limb showed an elongated intraneural swelling over the left sciatic nerve at mid-thigh level (**Figures 1C, D**). Other causes of neuropathy, such as infectious, inflammatory, or nutritional, were ruled out by relevant blood tests. An urgent MRI of the spine showed no compression of the cauda equina (**Figure 1E**). A nerve conduction study confirmed sensorimotor axonal neuropathy of the right ulnar and left sciatic nerves.

**Figure 2:**
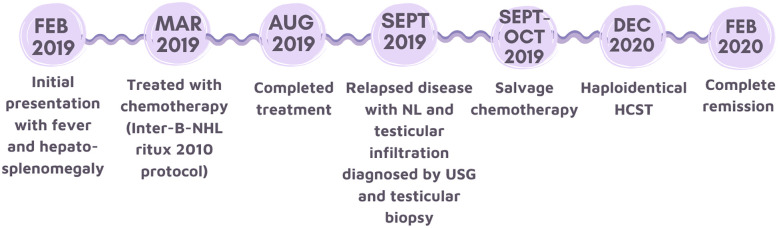
Timeline showing the sequence of events of this case.

Histology of trans-scrotal testicular biopsy confirmed relapse of high-grade B cell lymphoma. No nerve biopsy was performed. The patient was diagnosed to have NL of the right ulnar nerve and left sciatic nerve based on clinical and radiological features. Staging PET-CT scan showed increased FDG activity over the right mid-arm (**Figure 1F**) and left lower thigh, corresponding to the nerve lesions. Bone marrow aspirate and cerebrospinal fluid were negative for lymphoma cells. The patient was treated with salvage chemotherapy [rituximab, vincristine, ifosfamide, carboplatin, and idarubicin (R-VICI regimen)] (5). After two cycles of R-VICI, USG showed the resolution of ulnar nerve lesions and shrinkage of sciatic nerve lesions. The patient achieved good partial remission of the relapsed disease and proceeded to hematopoietic stem cell transplantation at about two months from the time of disease relapse. USG documented the resolution of the peripheral nerve lesions. The neuropathic pain gradually subsided with the weaning of the analgesics such as gabapentin. He could walk independently for 45 minutes six months post-transplantation and had normal hand function. His neurological deficits had improved significantly, with complete motor function recovery but with mild sensory deficits. His nerve conduction study performed 2 years later showed partial recovery of motor response in right ulnar nerve and left peroneal nerve as well as chronic neurogenic changes of left tibialis anterior and left gastrocnemius in electromyography study. The patient and his family showed great satisfaction with the recovery. His disease remained in continual complete remission for five years (**Figure 2**).

## Discussion

We present a case of sensorimotor neurolymphomatosis (NL) of the sciatic and ulnar nerves in a child with relapsed high-grade B cell lymphoma. Our case is unique, as it is the only pediatric case of peripheral neuropathy presented as the first symptom of lymphoma relapse. The onset of a new peripheral neuropathy in a cancer patient should raise suspicion on a diagnosis of NL, after ruling out more commonly occurring conditions such as neuropathy due to nutrition or chemotherapy. The role of different radiological modalities was introduced to guide pediatric oncologists in choosing suitable tests. Early identification of the clinical signs of peripheral neuropathy and the USG features allow us to diagnose NL efficiently and initiate treatment to salvage the case.

The mechanism of neurolymphomatosis is believed to be due to the expression of neural cell adhesion molecules on lymphoma cells (6). It was found that lymphoma cells can directly invade the endoneurium, which is uncommon in other malignancies (7).

There have been abundant adult case reports and case series on lymphoma infiltration into the nerves (8–14). To date, three cases of pediatric NL have been reported (1–3). A recently published systematic review on 459 NL cases and some adult case series (**Table 1**) showed that diffuse large B cell lymphoma is the most common lymphoid malignancy presenting with NL. Painful sensorimotor peripheral neuropathy is the most common neurological presentation. The gold standard for diagnosis of NL is nerve biopsy, which is not readily performed due to the risk of nerve injury. As an alternative, a histological diagnosis of other involved areas, such as lymph nodes or testis, as in our case, could confirm the diagnosis. Since NL is a rare entity and its presentation is non-specific, non-invasive imaging studies are thus very useful in guiding the diagnosis. USG is a readily available screening tool to identify and localize the abnormality, especially if the lesions are palpable. The presence of an intralesional color Doppler signal and an intraneural involvement point more toward a tumor infiltration into the nerves rather than other causes, such as drug-induced peripheral neuropathy. USG can also help with disease monitoring and guide biopsies. Although the literature did not show many cases of NL diagnosed by USG, a more recently published paper by Wada et al. confirmed the valuable role of USG as a screening tool and an evaluation method for disease response (15). On the other hand, MRI can further delineate the affected tissue and anatomical relationship. The relevant MRI findings include T2-weighted hyperintense signal, contrast enhancement, and linear or fusiform thickening of the involved nerves. Regardless of the imaging modality, the radiological findings should always be interpreted with the clinical presentation, laboratory findings, with or without a definitive histology.

**Table 1:** A literature review of case series with neurolymphomatosis (10, 17–20).

Author	Gender	Age (median)	Clinical feature	Nerve structure	Cancer type	Time of NL diagnosis	MRI	PET	USG	CSF	Nerve biopsy	Treatment	Neurology outcome	Overall survival (median)
Byun JM (17) (12 cases)	66.7% male	NA	66.7% painful neuropathies, 50% sensorimotor neuropathy	66.7% peripheral nerve, 42% nerve roots, 25% plexus, 8.3% cauda equina	75% DLBCL, 16.7% Burkitt, 8.3% ALCL	25% progression during treatment, 75% during relapse	88.9% +	90% +	NA	11.1% +	2 biopsies done: +	100% chemotherapy, 41.6% radiotherapy, 41.6% IT chemotherapy	16.7% improvement, 41.6% stable, 41.66% aggravation	4.5 months
Jeong J (18) (10 cases)	80% male	55 years	100% painful sensorimotor neuropathies	30% peripheral nerve, 40% plexus, 80% nerve roots, 10% cranial nerve	80% DLBCL, 10% mantle cell, 10% marginal zone	10% primary disease, 60% progression during treatment, 30% during relapse	88.9% +	100% +	NA	11.1%+	2 biopsies done: +	100% chemotherapy, 60% radiotherapy, 60% IT chemotherapy	30% improvement, 50% stable, 20% aggravation	5 months
Fritzhand SJ (19) (18 cases)	72.2% male	63 years	100% cranial neuropathies	100% cranial nerve	50% DLBCL, 16.7% marginal zone, 11.1% low-grade B cell, 11.1% mantle cell, 11.1% others	55.6% primary disease, 44.4% during relapse	100% +	NA	NA	NA	NA	100% chemotherapy, 11.1% radiotherapy, 5.6% HSCT, 5.6% IT chemotherapy	NA	NA
Khurana A (20) (40 cases)	60% male	60.5 years	70% painful neuropathies, 82% motor involvement, 80% sensory involvement	58% peripheral nerve,42% plexus, 35% nerve roots, 20% cranial nerve	68% DLBCL, 15% low-grade B cell, 25% others	52% primary disease, 48% during treatment or relapse	100% +	74%	NA	24%	100%	93% chemotherapy, 12.5% radiotherapy, 12.5% IT chemotherapy, 40% HSCT	77% improvement	72.6 months
Ducatel P (10) (9 cases)	66.7% male	71 years	78% painful neuropathies, 44% sensorimotor, 56% mononeuropathy	100% peripheral nerve	44% DLBCL, 11% mantle cell, 11% low-grade B cell, 33% others	NA	29% +	38% +	NA	0%	8 biopsies done: +	100% chemotherapy, 33% IT chemotherapy, 11% HSCT	12.5% improvement, 37.5% stable, 50% aggravation	NA

DLBCL, diffuse large B cell lymphoma; ALCL, anaplastic large cell lymphoma; NA, not available; IT, intrathecal; HSCT, hematopoietic stem cell transplantation; +, positive.

The prognosis of relapsed high-grade B cell lymphoma is poor, with a 4-year overall survival rate of ~15% (16). There is no consensus on the best treatment for neurolymphomatosis. We used rituximab combined with chemotherapy bridged with hematopoietic stem cell transplantation to achieve clinical remission in this case. However, most reported NL cases had a delayed diagnosis of lymphoma. The median overall survival of secondary NL quoted from Kaulen et al. was only 13 months (8). Therefore, it is imperative to identify the signs and symptoms of NL early, so we can initiate treatment promptly for this aggressive disease.

## Concluding remarks

In summary, NL may be the first presentation of an underlying lymphoid malignancy or recurrence of lymphoma. Targeted USG of the peripheral nerve followed by MRI or PET-CT will help to diagnose NL and exclude other diagnoses. Early diagnosis is critical, particularly in the context of relapsed aggressive B cell lymphoma, to facilitate early institution of salvage treatment.

## Data Availability

The original contributions presented in the study are included in the article/supplementary material. Further inquiries can be directed to the corresponding author.
